# Integrating Brain Science and Law: Neuroscientific Evidence and Legal Perspectives on Protecting Individual Liberties

**DOI:** 10.3389/fnins.2017.00621

**Published:** 2017-11-08

**Authors:** Calvin J. Kraft, James Giordano

**Affiliations:** ^1^Program of Liberal Studies, Neuroscience and Behavior, University of Notre Dame, Notre Dame, IN, United States; ^2^Departments of Neurology and Biochemistry, Pellegrino Center for Clinical Bioethics, Georgetown University Medical Center, Washington, DC, United States

**Keywords:** neurolaw, privacy, cognitive liberty, legal neuroscience, neuroevidence, neuroimaging, freedom of thought

## Abstract

Advances in neuroscientific techniques have found increasingly broader applications, including in legal neuroscience (or “neurolaw”), where experts in the brain sciences are called to testify in the courtroom. But does the incursion of neuroscience into the legal sphere constitute a threat to individual liberties? And what legal protections are there against such threats? In this paper, we outline individual rights as they interact with neuroscientific methods. We then proceed to examine the current uses of neuroscientific evidence, and ultimately determine whether the rights of the individual are endangered by such approaches. Based on our analysis, we conclude that while federal evidence rules constitute a substantial hurdle for the use of neuroscientific evidence, more ethical safeguards are needed to protect against future violations of fundamental rights. Finally, we assert that it will be increasingly imperative for the legal and neuroscientific communities to work together to better define the limits, capabilities, and intended direction of neuroscientific methods applicable for use in law.

## Introduction

“Mind reading” by government agencies is classic fodder for conspiracy theorists (see Constantine, 1995). But what makes this so evocative? The particular unease produced by imagination of such scenarios seems to be drawn from an intrinsic idea that the contents of thought are—and ought to be—private. Farahany (2012) has referred to these notions as “intuitions about mental privacy and autonomy of self” (354). The Supreme Court case Stanley v. Georgia (1969), among others, legally reinforced some of these intuitions, with Justice Thurgood Marshall stating that citizens should be “generally free from governmental intrusions into one's privacy and control of one's thoughts” (394 U.S. 557). Based on this doctrine, it would seem that thought is not criminalized in the American justice system. Yet this conclusion is complicated by the fact that the exploration of *mens rea* is a major pillar of due process. Terms like “intent,” “knowingly,” and “purposefully” figure prominently in courtroom decisions, and have alternately served to extenuate or implicate countless individuals (see Morissette v. United States, 1952 or Durham v United States, 1954). Thus a puzzling dialectic has evolved: on one side is the assurance that the courts have no business examining mental processes; on the other is that neuroscience is being increasingly used to generate inferences about private thoughts and motivations.

The resulting ethicolegal conundrum is a classic result of law incorporating insight from adjacent disciplines (see Goodenough and Tucker, 2010). In fact, the advent of “modern” neuroscience is only the latest instantiation of law encountering brain science. As Shen (2016) has shown, medical perspectives from early psychophysiologists have been influencing legal paradigms since the mid-nineteenth century (670); in particular, investigations into the material correlates of mental phenomena have instituted new schools of legal thought regarding definitions of guilt, criminal responsibility, and personhood (see Kolber, 2015; Morse, 2015). Perhaps the most visible example of law's incorporation of neuroscience has occurred in the past few decades—as the use of neuroscientific evidence in court becomes more and more common.

New techniques and tools of neuroscience now produce results purportedly indicative of the workings of cognitive processes, or what may commonly be regarded as the “mind”: whether it be recognition of a certain piece of information and/or a neural “signature” of having been in a certain place at a certain time; and whether or not one is engaging in deception (Fang et al., 2003; Davatzikos et al., 2005). These techniques remain tentative outside of laboratory settings, but still have triggered debates about the validity, ethicality, and legality of such procedures should they be introduced into the court (Keckler, 2005; Meegan, 2008). Currently, the use of neuroscientific evidence by the defense is somewhat in vogue; it is frequently used to assert diminished capacity, insanity, and/or plead for mitigation (Brown and Murphy, 2010; Farahany, 2016). This development has led to increased scrutiny around the effects of technological change on legal definitions of guilt (Greene and Cohen, 2004; see section Looking Forward). In contrast, the successful use of neuroscientific evidence by the prosecution to assert guilt remains lacking (Giordano et al., 2014b)—most likely because forensic methods in neuroscience have not yet met admissibility standards of the court (Alexander, 2007). This latter point brings to the fore a key issue: namely, that the orientation and goals of legal neuroscience are not yet clearly defined, and the law may need to clarify what exactly it is asking from neuroscience (Shats et al., 2016).

In this light, the focus of this paper can be summarized by a single question: *Does the modern use of neuroscientific evidence in criminal courts pose challenges to the rights of the individual?* This query may be broken down into three parts: (1) What are the rights of the individual *vis-à-vis* the acquisition and use of neuroscientific investigation? (2) Is (and how is) neuroscientific evidence currently accepted and used in criminal courts? and (3) Are the ethical injunctions established in (1) violated by uses outlined in (2)?

Regarding the rights of the individual, we will use the U.S. Constitution's Bill of Rights as primary reference, given that this document has become a benchmark for basic constructs of human rights in modern democratic societies. We also review a number of landmark court cases that have helped to elucidate how these rights have been interpreted. For the second question, we will inquire into the most viable neuroscientific techniques currently projected toward—or employed in—legal use. Finally, we address whether current protections are likely to persist despite technological change, and how the neuroscientific and legal communities can (and perhaps should) work to safeguard the rights of the individual.

## “Neuro-rights” in the modern world

What precedent is there for establishing the “neuro-cognitive” rights of the individual? Shen (2013) has commented that the growing capabilities of neuroscience have spurred a kind of “mental privacy panic” (668); in the wake, there have been numerous attempts to create a framework for protecting these rights. Boire (2001) and Greely (2006) were among the first to investigate how these rights are linked fundamentally to the human person. Kostiuk (2012) has suggested that current United States law inadequately protects the use of neurological information, and to that end has proposed legislation to establish a Neurological Information Non-discrimination Act (NINA), which is modeled on the Genetic Information Non-discrimination Act (GINA) of 2008. But NINA would only address potential misuse by employers and other private entities. How would individual “neuro-cognitive rights” be protected in the American courtroom? Many have looked to the Constitution of the United States as a foundation for these rights (see Boire, 2005; Tovino, 2007; Shen, 2013). Of course, the rights defined by the Constitution do not immediately seem to approximate intuitions about the sanctity of thought—and it is unlikely that the framers of the Constitution were prepared to consider mental processes as being externally assessable. Yet a possible framework for the preservation of what Boire has called “cognitive liberty” (2001) may be found by interpretation of key aspects of the Bill of Rights.

### First amendment: the “double aspect”

The First Amendment does not seem to allow for protection of the workings of the mind, but rather affords rights for the outward expression of those processes. Yet, in Jones v Opelika (1942), the court inextricably linked the two, writing that “freedom of speech, freedom of the press, and freedom of religion all have a double aspect—freedom of thought and freedom of action” (316 U.S. 618). A similar argument is expressed in Palko v. Connecticut (1937), in which Justice Cardozo asserted that “freedom of thought and speech” is the “matrix, the indispensable condition, of nearly every other form of freedom” (302 U.S. 327). It is notable that these decisions assert not only that thought *should be* inviolable, but that it inherently *is*—for instance, the *Opelika* decision claims that “freedom to think is absolute of its own nature; the most tyrannical government is powerless to control the inward workings of the mind” (316 U.S. 618). Techniques for (blunt) external modulation of cognitive processes—ranging from the pharmacological (Wright, 2005) to the surgical (Faria, 2013)—were available even then, and seem to belie the notion that processes and mechanisms of thought are untouchable. As such, the relevance of these decisions is called into question, especially in light of today's increasingly sophisticated and non- (or at least minimally) invasive technologies (e.g., transcranial magnetic and/or electrical stimulation; see Narayana et al., 2017).

The aforementioned invocations of the First Amendment only apply to government *interference* with cognitive processes, rather than mere *observation* (Tovino, 2007), so perhaps in this case the First Amendment lacks precision and therefore value in engendering fair protection. Still, it might be successfully argued that the mere knowledge of the judicial use of neuroimaging could exercise a “chilling effect” on the freedom of thought, just as phone conversations might become more guarded in a country where wiretapping is legal and widespread. This is especially true if individuals begin to consider that their thoughts might later serve to incriminate them.

### Fourth amendment: protecting privacy

The Fourth Amendment has long been used to justify the preservation of a personal, private sphere where government may not intrude. Schmerber v. California (1966) asserted that “the overriding function of the Fourth Amendment is to protect personal privacy and dignity against unwarranted intrusion by the State” (384 U.S. 767). In the case of collecting neuroscientific evidence, it is the amendment's “search and seizure” clause that is most relevant (Boire, 2005; Shen, 2013). The question is whether gathering neuro-cognitive evidence constitutes an overly intrusive search of an individual's person. For instance, in Winston v. Lee (1985), the court argued that an invasive procedure, like surgery, violates reasonableness standards established by prior decisions (470 U.S. 753). The court also rejected warrantless searches of the kind conducted in Kyllo v United States (2001), in which a thermal-imaging device was applied to a suspect's home (533 U.S. 23); this kind of device, which gathers information about the internal state of a private space without physical intrusion, might be analogized to certain forms of neuroimaging (Boire, 2005). Boire uses this case to argue that a “head” should have even more protections than a “home,” and that informed consent—not just a warrant—should be a requirement for any scans (i.e., “searches”) of the brain.

Recently, rights guaranteed by the Fourth Amendment have been curtailed in significant ways. In 2013, the court ruled in *Maryland v King* that collection of DNA samples upon arrest is a constitutional procedure (133 S. Ct. 1958). That case further reinforces the notion from *Schmerber* that not all biological evidence is sacrosanct (384 U.S. 757). But it also includes the justification that since DNA samples can help determine an individual's criminal history, their collection serves “legitimate government interest” in determining whether an individual might pose a danger to society (133 S. Ct. 1959). This argument seems to open the door for other procedures that would establish the level of danger an individual poses to society; procedures that might eventually incorporate neuroimaging and perhaps other forms of neuro-cognitive assessment. Such decisions seem to reflect a desire to balance the rights of the individual with concerns about the safety of the society (for further discussion, see Giordano et al., 2014b; Giordano, 2015a). Nowhere is this trend clearer than in the institution of the Patriot Act in the United States after the attacks of September 11, 2001, when privacy expectations from the Fourth Amendment seemed to be superseded by an overarching imperative for public safety (Osher, 2002). Might changing standards in modern society impact notions of what constitutes an acceptable reason for invasion of privacy, and could this impact the criminal courts?

### Fifth amendment: against self-incrimination

In one of the more famous court decisions of US history, (Miranda v Arizona, 1966), the courts ruled that an individual has the “right to remain silent,” in order to uphold the “privilege against self–incrimination […which] is the essential mainstay of our adversary system” (384 U.S. 460). With this decision, the courts were drawing upon centuries of legal scholarship with roots in Hobbes' “right of nature” (1651), which contended that each person is naturally free to act in their own self-interest. This right is enshrined in the Fifth Amendment's provision that no person “shall be compelled in any criminal case to be a witness against himself.”

The legal definition of “self-incrimination,” however, has proven to be somewhat ambiguous. One early challenge to the principles of *Miranda* came in *Schmerber v. California*, in which the defendant was compelled to provide a blood sample to verify his level of intoxication (384 U.S. 757). The court ruled that the use of this evidence, although taken from the defendant's own person, did not constitute a violation of the Fifth Amendment protections, because the evidence was not “testimonial or communicative” in nature (384 U.S. 761). In that same decision, the court remarked that “to compel a person to submit to testing in which an effort will be made to determine his guilt or innocence on the basis of physiological responses, whether willed or not, is to evoke the spirit and history of the Fifth Amendment” (384 U.S. 764). While this assertion was ostensibly directed toward the contemporary use of the polygraph, it seems to have serious implications for use of (newer, valid, and more accurate) neuroscientific methods of deception detection in the courtroom.

### Fifth and fourteenth amendments: due process

One of the most fundamental provisions in the Bill of Rights is the “due process” clause of the Fifth and Fourteenth Amendments; it assures the right to a fair trial and stipulates that no individual may be convicted or otherwise deprived of “life, liberty, [or] property” without proper procedure. The “due process” clause has been flexibly interpreted to incorporate basic principles of common law, which include stipulations about the kind of evidence that should be admissible in court. For instance, in State of New Jersey v. Michaels (1994), the court explained that it “has a responsibility to ensure that evidence admitted at trial is sufficiently reliable so that it may be of use to the finder of fact who will draw the ultimate conclusions of guilt or innocence. That concern implicates principles of constitutional due process” (136 N.J. 316). That same decision cited (Manson v Braithwaite, 1977), which declared that “reliability is the linchpin” in determining the admissibility of evidence (432 U.S. 98). Such concerns about the probative value of evidence seem to be central to the maintenance of a fair and functional legal system.

It is by this foundational principle that the edifice of evidence law is justified, along with the landmark decisions of Frye v United States (1923, 293 F. 1013) and *Daubert v Merrell Dow Pharmaceuticals* (1993, 509 U.S. 579). These two decisions are especially relevant in the case of neuroscientific evidence, because they established a framework by which the admissibility of expert testimony in this field might be judged. While this framework is not completely standardized across state lines, it has been codified at the federal level in the revised Rule 702 (Bernstein and Jackson, 2004; Shats et al., 2016). Its stipulations regarding the relevance, validity, and reliability of scientific evidence are essential in the preservation of due process (see Keckler, 2005; Alexander, 2007).

In another invocation of due process, some have argued that neuroscientific evidence has a tendency to exercise an undue influence on juries out of proportion to its probative value (McCabe and Castel, 2008; Brown and Murphy, 2010). In such cases, Brown and Murphy (2010) argue that Federal Rule of Evidence 403 should be applied, which provides against “unfairly prejudicial” evidence (1188). However, others have contended that there is “little empirical support” (716) for claims that neurological evidence is inherently prejudicial (Farah and Hook, 2013; Shats et al., 2016).

## Neuroscience in legal contexts

We now briefly review two examples of neurotechnology currently being oriented toward legal use—specifically, for deception detection. While these techniques do not constitute the majority of neurological evidence being used in today's courts, they are unique for the potential challenges they may pose to individual rights.

### ERP: recognition and “guilty knowledge”

One possible source of neurological evidence in the courtroom is from information obtained through the use of event-related potentials (ERP) measured by electroencephalography (EEG). Most of these techniques center on the detection of “guilty knowledge” via P300 recognition signatures (Rosenfield et al., 1988). Essentially, the test relies on a well-documented neurological response to recognized information, which might be used to determine whether an individual has intimate knowledge about a crime (Rosenfield et al., 1988). P300 markers have been a staple of neurophysiological research for over half a century (Sutton et al., 1965); this, along with the fact that such markers appear reliably across subjects, represents this method's primary strength as a potential legal tool.

However, the regular appearance of such P300 markers does not mean that their functional correlates are well understood. As Meijer et al. (2012) noted, P300 responses have been identified with a range of responses that are similar only in their violation of expectation; this hardly denotes the functional specificity of an unambiguous “deception detector.” As such, ERP techniques often engender many of the same issues as other forms of “lie detectors,” which suffer from a lack of—or excessive ambiguity in—direct connection between the physical response and its interpretation (Meijer et al., 2012). It is also unclear if these methods are as reliable in the field as they are in the laboratory (Wolpe et al., 2005).

A form of ERP analysis that can be used for deception detection has been patented and marketed as “brain fingerprinting” (Farwell and Donchin, 1991; Farwell, 2012). While promising, this method has received censure from some in the scientific community for lack of transparency and overstatement of effectiveness (Wolpe et al., 2005; Meijer et al., 2012). To date, the use of this technology has made meager incursions into the legal system—for example, the case of *Harrington v Iowa* (2003; see Farwell *amicus curiae* brief of 2002)—but is typically discounted by judges for its lack of general acceptance (Wolpe et al., 2005).

### fMRI: descriptive challenges

Because functional magnetic resonance imaging (fMRI) provides a three-dimensional image of both cortical and sub-cortical activity of the brain (unlike the summative cortical responses assessed by EEG), it has greater descriptive potential. However, fMRI is also constrained by practical parameters (e.g., speed, variable correlative reliability of individual to group and group to individual comparisons) that impair its descriptive capability and power (Wolpe et al., 2005; Logothetis, 2008). Rusconi and Mitchener-Nissen (2013) have noted that “nearly every article” expounding on the potential of fMRI lie detection techniques has a section about “*issues yet to be resolved*” (594). Nevertheless, these issues have not prevented the emergence of commercial entities purporting to provide reliable fMRI-based deception detection (Cephos^1^; No Lie MRI, Inc.^2^).

Modern studies of deception detection using fMRI technology rely on subtle changes in the blood-oxygenation level of specific areas in the brain, generally, the fronto-parietal lobes and loci and networks of the limbic system (Hakun et al., 2009; Rusconi and Mitchener-Nissen, 2013). Such studies often claim accuracy rates of ninety percent or greater (Rusconi and Mitchener-Nissen, 2013; for an example, see Vartanian et al., 2012). These studies, however, are conducted under controlled conditions, with willing and reasonably relaxed subjects. Actual conditions in the context of a criminal investigation are likely to vary widely from those of the laboratory setting; such use would be further complicated by the fact that fMRI deception detection can be intentionally countered by “experienced” individuals with intent to deceive (Ganis et al., 2011). Wolpe et al. (2005) have noted that while within-subject reliability might be high, the true indicator of fMRI's value as a deception detector is its predictive power for use and applicability in the wider population—that is, its ability to determine whether a *particular* individual is lying, by using data obtained from previous subjects. Finally, Spence (2008) noted that experimental paradigms vary widely among contemporary peer-reviewed studies, with differing methods that employ differing tools, such as guilty knowledge tests, reviews of episodic memory, malingered memory impairment, etc.

As a result of these concerns about validity and reliability, fMRI-based deception detection has been used less frequently than ERP-EEG (Brown and Murphy, 2010; Rusconi and Mitchener-Nissen, 2013). Perhaps the most high-profile legal use of fMRI for deception to date is the case of United States v Semrau (2012), in which the defendant tried to introduce supposedly exonerating evidence performed by a company specializing in fMRI-based deception detection; the court ruled that the evidence failed to meet the standards of general acceptance and known error rates outlined by *Daubert* (14–16), and it was disallowed from the proceedings (Miller, 2010). Nevertheless, these early stumbles in judicial use of fMRI have not been altogether discouraging. Bles and Haynes (2008) acknowledged formidable obstacles while also carefully describing field studies that could increase the external validity of fMRI-based techniques (89–90). Langleben and Moriarty (2013) conceived of a “public funding initiative” and “peer-reviewed translational research program” that might provide the impetus to introduce rigor and widespread acceptance of fMRI deception detection techniques (231). And Hyman (2010), writing 5 years after the seminal paper by Wolpe et al. (2005), noted that computational analysis of fMRI data had made great strides since the publication of their earlier appraisal of the field.

### Other uses

The most common current employment of neuroscience in the courts is not related to deception detection, nor to any kind of forensic application; instead, the most frequent use of neurological evidence is by the defense (Shats et al., 2016), usually arguing for diminished capacity, insanity, or pleading for mitigation (Seiden, 2004; Farahany, 2016; Shen, 2016). Indeed, some have noted that the presentation of structural brain scans (i.e., to demonstrate neurological abnormalities that might have influenced the commission of the crime) at sentencing is “now almost invariably present in capital cases” (Rusconi and Mitchener-Nissen, 2013). Since such evidence is intended ostensibly to support the defendant's case, it is unlikely it would represent a threat to individual liberties as long as valid, reliable techniques and technology are used.

### Liberties at risk: freedom of thought

As we have previously noted, “freedom of thought” as described by the courts seems to refer strictly to the unfettered exercise of thought without fear of external interference or punishment. Since the ostensible goal of deception detection technology is to determine what an individual has *done*, and the US legal system is not structurally oriented toward punishing individuals for their thoughts alone (see the court's formulation of *mens rea* in Morissette v. United States, 1952), it does not seem that this particular sense of cognitive liberty is directly violated by the current developments in neuroscience.

But are challenges to freedom of thought looming in the near future? Modern techniques do not seem to be apprehending “thought” by its typical definition (see Illes, 2007), and neurotechnology is unlikely to have a “chilling effect” on freedom of thought until it is able to definitively link observable brain states with complex cognitive processes. Along these lines, Gazzaniga (2005) has argued extensively against what he calls a “slippery-slope” fallacy that equates techniques like detection of (face or object) recognition with mind reading (xvii). Similarly, Meegan (2008) has likened guilty-knowledge technology at its best to a black-box camera that only indicates when a duplicate picture has been taken (16). Logothetis (2008) has stated somewhat more bluntly that: “…fMRI is not and will never be a mind reader” (869).

Yet this final claim may be called into question by a more integrative approach to development and use of neuroscientific techniques and technologies, as well as a more detailed philosophical examination of what the concept of “mind reading” really entails and obtains. In the past decade, advances in systems and computational neuroscience have enabled increasingly detailed reconstruction(s) of subjects' visual experience (Nishimoto et al., 2011), as well as description of the semantic content of viewed images (Huth et al., 2016), and prediction of narrative thinking (Wang et al., 2017) based on fMRI scans of various regions and networks of the brain. The technology seems to be toeing a line that many had considered unreachable. So, when we say that someone is “reading our mind,” do we necessarily mean that our thoughts are being understood in the exact form that we subjectively experience them? Or perhaps more appropriately, is it more realistic (and perhaps valuable) to consider reading in the literal sense: as “apprehend[ing] meaning by perceiving…form and relation…interpret[ed] in a specified manner determined by consensus” (Funk and Wagnall's, 1967)? By that definition, neuroscience may be poised at—and moving closer toward—the threshold of at least rudimentary “mind reading” capability. Given such advancements, might even a fuzzy approximation of the visual and/or semantic content of thoughts (with a known error rate) violate constructs of cognitive autonomy? New methods in neuroscience are both re-defining concepts of consciousness, as well as providing ways that subjective experience can be accessed, obtained and interpreted by others (Evers and Giordano, in press). Kolber (2014) has also noted the state's significant powers of subpoena in regard to memories. The question then arises: to what ends?

### Invasions of privacy

So while acquisition of neurological evidence cannot yet be likened to a “search” of an individual's thoughts, the capability to perform such a search is waxing into the realm of possibility—and the court has done little to concretely define the private sphere as it relates to neuro-cognitive liberty. What precedent there is regarding the use of private papers as evidence is tenuous and inconsistently applied across circuit courts (Farahany, 2012, p. 384). A substantive challenge to Fourth Amendment protections is suggested by the recent Maryland v King (2013) decision. If “legitimate government interest” in the safety of society serves as a justification for collecting DNA upon an individual's arrest, might a cursory neuroimaging scan to detect potentially dangerous anomalies of structure and/or function be similarly justified? In this scenario, the widespread references to frontal lobe damage by defendants to argue for sentence mitigation may be indirectly curtailing individual rights.

In a similar vein, Wolpe et al. (2005) suggested a hypothetical situation that might trigger Fourth Amendment protections, where “imaging for a non-medical indication could reveal medically relevant information” (46; see also (Wolf et al., 2008) for more commentary on incidental findings). For example, if a defendant willingly subjects her/himself to an fMRI deception evaluation, as in *Semrau*, and the fMR images reveal a brain tumor, are the images (still) admissible in court? Such problems are inherent to a technology that provides more information than is warranted by the legal system.

### Self-incrimination

The determination of whether neurological evidence constitutes a violation of Fifth Amendment protections hinges on the classification of such evidence. As Farahany (2012) has argued, *Schmerber* established precedent for a tendentious dichotomy between “physical” or “biological” evidence and “testimony”. In its place, she proposed a spectrum of evidence, beginning with “identifying” information like height and appearance, and ranging through “automatic,” “memorialized,” and “uttered” domains (368–389). Where would neurological information fall on the spectrum? Given that complex ideas and semantic information cannot currently be reliably detected by available neurotechnology, neurological evidence probably would not be in the “uttered” category. Deception detection methods would produce evidence in the “memorialized” category, while basic structural information about an individual's brain would fall in the “automatic” category. Using this approach, she concluded that the extant legal framework protecting against self-incrimination would produce a result that is “deeply unsatisfying and at odds with ordinary intuitions about mental autonomy” when applied to memorialized neurological evidence (389).

Farahany's argument might be considered conjectural, since the only current instances of neurological deception detection in the United States have been conducted with the consent and cooperation of the subject. However, societies do not make identical judgments, and India has reportedly embraced court-mandated ERP-based deception detection techniques (395; Ghiridharadas, 2008). In the United States, the advancement of neurologically-based deception detection in legal contexts has heretofore been largely halted by evidence law (see following section), but recent advancements approaching the “horizon of potentiality”^3^ might make it prudent to create more stringent ethico-legal safeguards, perhaps along the lines of the French Civil Code, which has recently been updated to stipulate that any neuroimaging must occur with the express consent of the individual, which may be revoked at any time (Article 16–14, as noted by Rusconi and Mitchener-Nissen, 2013). A possible route for requiring informed consent for deception detection techniques may be analogized from the current admissibility standards for the traditional polygraph in the United States. While United States v Scheffer (1997) set a precedent for excluding polygraphic evidence in federal courtrooms, the states have inconsistently followed its example: twenty-nine states have banned such evidence outright, but the remaining states do not exclude it *per se*, provided that both the prosecution *and* the defense consent to its use (Shniderman, 2012). However, despite recent calls for ongoing protection of individuals who consent to the use of novel neurotechnologies, and the information they may yield (Giordano, 2015a,b,c, 2017), at present there are no policies in place that would provide for such safeguarding (thereby fortifying the importance and need for some form of “NINA”; see above).

### Due process

We have demonstrated how the use of current neurotechnology may be constrained in producing valid and reliable inferences about a defendant's cognitive processes, but also have noted that commercial entities exist for apparently detecting such processes in judicial settings. To preserve due process, then, we posit that a reliable “gatekeeper” is needed. The aforementioned *Daubert* and *Frye* standards have proven adequate to the task (Brindley and Giordano, 2014). As an early example, in People of NY v Weinstein (1992), a judge cited the *Frye* standards when disallowing expert testimony that an arachnoid cyst near the frontal lobe may have caused the defendant to murder his wife, stating then that it was not “generally accepted” that such cysts are responsible for acts of violence (156 Misc.2d. 34). A more recent example of gatekeeping in action is the previously mentioned United States v Semrau (2012), in which fMRI-based deception detection was excluded from the case on grounds that the technology was untested in “real world settings,” pursuant to FRE 702 (6; Miller, 2010).

Based on cases like these, it has been concluded that the *Daubert* standard (and related guidelines) present a formidable challenge to legally-oriented uses of neurotechnology. Keckler (2005) and Alexander (2007) extensively reviewed neurological deception detection and affirmed that, barring future (and significant) advances in validity and reliability, such evidence would likely continue to be excluded from legal consideration. Shats et al. (2016) came to a similar conclusion, while providing caution that the character of neurologically-based evidence requires that “the court… be particularly vigilant to ensure that the experts are being asked the appropriate questions, and that… they remain within the confines of the role of expert witnesses” rather than acting as judge or jury in themselves (722). With the possible exception of an unduly prejudicial effect from neuroscientific evidence, the current iterations and use of neurotechnology do not appear to threaten due process.

### Looking forward

But with technological advancements and shifting cultural perceptions, will the rights outlined here face greater threat in the future? For their part, the rules of evidence, especially at the federal level, will most probably continue to preserve due process to the extent that they have since the *Daubert* case. Their stipulations continue to align well with basic principles of “good” science, and will reject inadequate methods and technologies (e.g., of neuroimaging and/or neurogenetics) just as effectively as Frye v United States (1923) rejected specious blood pressure data nearly a century ago (293 F. 1013). Barring any fundamental changes in the philosophy of science, the integrity of evidence and court proceedings in general are duly preserved by the gatekeepers of the court. Yet, if (and arguably when) forensic neuroscience eventually meets the standards for admission of evidence, it is unclear if other rights will be so carefully safeguarded. Technological advances in the past decade suggest that the challenges to reliable detection of mental states are not as insurmountable as previously regarded. More than ever, the inviolability of the mind as outlined in *Palko v Connecticut* and *Jones v Opelika* appears to be a moral, rather than a natural, imperative.

Even the “intuitions” described by Farahany (2012) might not be immune to societal pressures. Kittay (2007) posited that the most significant hurdle to the use of fMRI for deception detection might not stem from legal or technological issues, but rather from negative public sentiment toward “mind reading devices.” But with continuing developments and use of such neurotechnologies, and the somewhat ubiquitous, although not always accurate presentation of neuroimaging findings in the public eye, might a gradual acceptance of such techniques leach into public perceptions? Numerous neuroethicists have argued that cultural expectations could shift—or might already be shifting—amidst a preponderance of neuroscientific information: in definitions of personhood, guilt, and free will (see Greene and Cohen, 2004; Santosuosso and Bottalico, 2009). One of the most significant sources of this type of change may be the neuroscientific evidence already in courts—that is, the use by the defense described by Farahany (2016). Employment of this tactic might not only lead to a normalization of neuroscience in the courtroom, but could also cause deterministic paradigms to leach into legal conceptions of culpability (see Greene and Cohen, 2004; Mobbs et al., 2007). It is unclear if traditional rights and liberties would still be relevant in the deterministic plane—though some assert that social intuitions about guilt are little affected by metaphysical conditions (Roskies, 2006; Nahmias et al., 2014). Another change in cultural perceptions might be revealed by the recent trend favoring collective benefit over individual privacy, as demonstrated by *Maryland v King*, the Patriot Act, and the rising calls for, use and discussions of “big data” (Giordano et al., 2014b; DiEuliis and Giordano, 2016).

Finally, although equating any modern technology to a “search of an individual's thoughts” might be an overstatement, the court has yet to address significant ambiguities in its definition of the private sphere and what exactly constitutes “overly invasive” use of neuroscientific tools and techniques. assessment Farahany's (2012) of the *Schmerber* guidelines also elucidated vulnerability in the Fifth Amendment protections, namely, the inadequacy of the physical/testimonial dichotomy. As previously mentioned, the court has also yet to explicitly address the matter of informed consent for the gathering of neurological data—and with the new standard of *Maryland v King*, the only limitations to compulsory brain scans upon arrest seem to be technological.

### What can be done?

We assert that such vulnerabilities should prompt the legal and neuroscientific communities to take action. On the side of the courts, the decision must be made as to whether the vulnerabilities described in the previous section are to be treated casuistically, as per tradition, or perhaps more prescriptively, as in the case of France's updated Civil Code. Additionally, judges and lawyers should remain abreast of developments in neuroscience and technology so as to properly consider admitting or excluding neurotechnologically-derived evidence (see Goodenough and Tucker, 2011). This does not mean, as Justice Rehnquist suggested in his dissent to the *Daubert* decision, that judges are required to become “amateur scientists” (509 U.S. 600), but rather that they should foster a working relationship with subject matter experts in neuroscience, in order to maintain an understanding of the limits and capabilities of contemporary tools and methods of brain science.

This also invokes obligations of the neuroscientific community: to be aware of the potential use (and misuse) of the techniques that are being developed; to develop an understanding of the legal and ethical limitations on broader applications of brain science; and finally (although not at all least), to work to avoid misrepresentation of neurotechnological capabilities to the public (Morse, 2011; Giordano, 2012a). Commercial ventures in neuroimaging and neurogenetics, while seemingly inevitable in a capitalist system, can be problematic in that they may foster public misconceptions about the state of the field. The potential of neurological evidence to have an “undue influence” on a jury (Brown and Murphy, 2010; Shats et al., 2016) is partly due to such sensationalism on the part of popular science. Neuroscience must not succumb to such aggrandizement if it is to figure legitimately in judicial proceedings.

As neuroscientists and legal scholars respond to changing technology and work increasingly in tandem, there will inevitably be growing pains as the principles and methods of science are confronted with the different standards of the legal paradigm (see Garland and Glimcher, 2006). Therefore, it is important that these experts become well aware of such differences and philosophical misalignments. For instance, it is not the role of the neuroscientist as expert witness to offer an opinion regarding the guilt or innocence of an individual, and to do so is to violate the legal prerogative of the jury (see General Electric Co. v. Joiner, 1997 and *US v Scheffer*). Rather, expert testimony should simply provide evidence that will assist the judge and/or jury in their final decision (Jones et al., 2013; Shats et al., 2016). Jones et al. (2013) noted “…science [is] about facts and law [is] about values” (731). This means that in law, unlike in science, “there are no maybes” (731), and that a court must reach a decision within a reasonable period of time in order to fulfill its obligations as a resolver of disputes. But if neuroscience is to be used in legal proceedings (and there is both a “push” and “pull” for such use; Giordano, 2012a, 2015a), then it is—and will be evermore—important to be aware of the changing capabilities of neurotechnology, and the demands presented (Goodenough and Tucker, 2010). In short, we believe that the question should not be “what can current neuroscience and neurotechnology do for law?” but rather, “what will the law demand of new developments in neuroscience and neurotechnology?” (Giordano et al., 2014b; Shats et al., 2016). In this light, the use of a directed, advanced integrative scientific convergence (AISC) approach might aptly meet the needs defined and called for by the law (Giordano, 2012b).

## Conclusion

Until neurotechnology can validly link brain states with complex ideas and experiences, there are three main threats to individual rights that arise from the use of neurological evidence: (1) the ambiguous definition of the private sphere; (2) the lack of clarity in the *Schmerber* dichotomy; and (3) the lack of guidelines on informed consent for the use of neurological evidence. Less clear but equally pertinent is the effect of use of such evidence by the defense on legal definitions and social intuitions of guilt. In reaching this conclusion, we have not developed a conception of individual “neuro-cognitive rights” beyond those outlined in the US Constitution, nor have we thoroughly examined the state of affairs in countries other than the United States. These ventures require a broader philosophical outlook, extensive research in international law, and have been well engaged by others (see Church, 2011; Spranger, 2012; Picozza, 2016).

Scientific methods and social norms are constantly changing. And it may be that such international perspectives, discourses, guidelines and laws will need to be considered and engaged if and as brain science continues to be advanced, and to be proposed for use in legal processes. Perhaps then the integrative approach should encompass not only a scientific and ethical effort (Giordano, 2012b), but a focal orientation toward defined applications in the courts in order to “explicitly orient and align the capabilities of the brain sciences with the goals and limitations of the law” (Shats et al., 2016, p. 723). For while Justice may be blind, she must remain vigilant, in order to insure that novel technology does not circumvent the mandates of law, and that law does not misconstrue the capabilities and/or limits of technology.

## Author contributions

CK and JG conceived and developed the idea for this manuscript; CK and JG wrote and reviewed the manuscript and CK and JG are responsible for its content.

### Conflict of interest statement

The authors declare that the research was conducted in the absence of any commercial or financial relationships that could be construed as a potential conflict of interest.

## Footnotes

^1^http://cephosdna.com; accessed 27 July 2017

^2^http://noliemri.com; accessed 27 July 2017

^3^The “horizon of potentiality” refers to a perspective of current-to-future developments (for example, in science and technology) in which the span from the present to the midpoint to the horizon represents the zone of probabilities, and that from the midpoint to the horizon represents the zone of possibility, which together create the [shifting] cusp of capabilities that form and define the horizon (Giordano et al., 2014a).
